# Registered care home managers’ experiences of responding to the national care home visiting guidance in England during the Covid-19 pandemic; a multi-method qualitative study

**DOI:** 10.1186/s12877-023-03935-w

**Published:** 2023-04-19

**Authors:** Josie Dixon, Klara Lorenz-Dant, Edmund Stubbs, Karen Harrison Dening, Manna Mostaghim, Daniel Casson

**Affiliations:** 1 Care Policy and Evaluation Centre, London School of Economics and Political Science, Houghton Street, London, WC2A 2AE UK; 2 University of Augsburg, Universitätsstraße 2, 86159 Augsburg, Germany; 3 De Montfort University, The Gateway, Leicester, LE1 9BH UK; 4 Dementia UK, 7th Floor, One Aldgate, London, EC3N 1RE UK; 5 Care England, 2nd Floor, 2 Devonshire Square, London, EC2M 4UJ UK

**Keywords:** COVID-19, Pandemic, Social care, Long-term care, Care home, Care home visiting, Family carer, Dementia, Multi-method, Qualitative, Thematic analysis

## Abstract

**Background:**

Visiting restrictions in care homes in England and many comparable countries during the Covid-19 pandemic were extensive and prolonged. We examined how care home managers experienced, understood and responded to the national care home visiting guidance in England in developing their visiting policies.

**Methods:**

A diverse sample of 121 care home managers across England, recruited through varied sources including the NIHR ENRICH network of care homes, completed a 10-item qualitative survey. Follow-up, in-depth qualitative interviews were conducted with a purposive sub-sample of 40 managers. Data were analysed thematically using Framework, a theoretically and methodologically flexible tool for data analysis in multiple researcher teams.

**Findings:**

Some viewed the national guidance positively; as supporting the restrictive measures they felt necessary to protect residents and staff from infection, or as setting a broad policy framework while allowing local discretion. More commonly, however, managers experienced challenges. These included the guidance being issued late; the initial document and frequent, media-led updates not being user-friendly; important gaps, particularly in relation to dementia and the risks and harms associated with restrictions; guidance being unhelpfully open to interpretation while restrictive interpretations by regulators limited apparent scope for discretion; fragmented systems of local governance and poor central-local coordination; inconsistent access and quality of support from local regulators wider sources of information, advice and support that, while often valued, were experienced as uncoordinated, duplicative and sometimes confusing; and insufficient account taken of workforce challenges.

**Conclusions:**

Underlying many of the challenges experienced were structural issues, for which there have been longstanding calls for investment and strategic reform. For increasing sector resilience, these should be urgently addressed. Future guidance would also be significantly strengthened by gathering better data, supporting well-facilitated peer exchange, engaging the sector more fully and dynamically in policy-making and learning from care home managers’ and staff’s experiences, particularly of assessing, managing and mitigating the wider risks and harms associated with visiting restrictions.

## Background

During the first wave of the Covid-19 pandemic, care home mortality rates were higher than in other settings; this trend was international [1], although UK rates were higher than most European countries [2]. During the first 10 weeks, death rates in care homes in England and Wales more than tripled, compared to deaths at home and in hospital, which increased by just 77 and 90 per cent respectively [3]. Similarly, the age-standardised mortality risk for people aged 65 and over in England, increased from 10 times higher in care homes than in other settings in February 2019 to 17 times higher in April 2020 [4]. Reasons include residents’ age, frailty and comorbidities, congregate living and frequent, close contact with caregivers [5, 6]. An additional contributing factor in England was that around 25,000 hospital patients were discharged into care homes without testing for Covid-19 over March and April 2020 [2, 7]. In a context of falling rates of Covid-related serious illness, disparities between settings narrowed over later waves, likely reflecting early vaccination programmes and improved infection control [1, 2].

In common with many other countries and reflecting care home residents’ greater vulnerability, the UK Government introduced visiting restrictions in care homes as a key infection control measure [8]. On 13^th^ March 2020, it advised against visiting a care home if feeling unwell^1^ and some, particularly larger, providers ceased all non-essential visits at this time [9]. The first national lockdown began on 23^rd^ March 2020, with a phased easing of restrictions occurring over subsequent months. While no formal ban on visits to care homes was issued over this time, initial advice was for no visitors except in exceptional (usually end-of-life) situations [10]. Care home providers and managers concerned about whether, when and how to re-introduce visiting into their homes as the nation moved out of lockdown were reliant on limited sector-led guidance, notably from the Care Provider Alliance (led by the National Care Forum)^2^ and the British Geriatrics Society,^3^ until comprehensive Government guidance on care home visiting was eventually issued on 22nd July 2020.

This guidance required that care homes develop *‘a policy for limited visits’* to be *‘made available and/or communicated to residents and families.’* Care homes’ visiting policies were to be based on a *‘dynamic risk assessment’* and advice from the local director of public health (DPH), as well as additional advice offered by the local clinical commissioning group (CCG)^4^ infection control lead and the local Public Health England^5^ health protection team (HPT). The guidance advised a single constant visitor, social distancing, use of personal protective equipment (PPE) and that visits take place outdoors, behind plastic/glass barriers, in a designated visitor room or, potentially, a resident's room. Care homes should rapidly increase restrictions (usually no visitors with scope for exceptional end-of-life visits) in the case of an outbreak, defined as lasting 28 days after the last suspected or confirmed case.

**Fig. 1:**
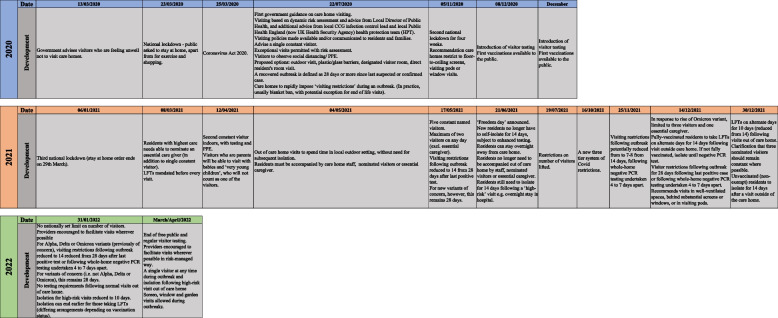
Care home visiting policy in England: Timeline

Numerous updates to the Government care home visiting guidance were made subsequently. Key changes (in England) are summarized in Fig. 1. The background to these changes included two further national lockdowns in November 2020 and January 2021, a tiered system of local restrictions from October 2021 and a public vaccination programme from December 2020. Visiting guidance updates involved changes to the number of visitors permitted, visitor testing from December 2020, the introduction of an essential caregiver role (designated family carer providing direct care who observes staff protocols and can visit any time) from March 2021 and various changes to the length of time and circumstances in which self-isolation is needed following visits out of the home. The visiting guidance was withdrawn in March 2022 with key measures subsumed into other infection control guidance for adult social care settings [11].^6^

Visiting restrictions were thus extensive and prolonged, with concerns expressed by researchers and others about associated harms for residents (e.g., loneliness, mood disorders, behavioural symptoms related to dementia, increased antidepressant and antipsychotic prescription, and loss of function) and families (e.g., feelings of guilt, fear, stress, and worry) [12, 13]. Also, while public health emergencies may merit proportionate limitations to human rights [14, 15], some argued that visiting restrictions insufficiently respected rights to liberty and a private and family life [10, 13, 16].

A range of research about experiences in care homes during the pandemic has been undertaken, much rapidly conducted to provide early input into policy, commonly using small-scale convenience samples [17, 18], with initial studies tending to focus on the experiences and perspectives of family carers, including their views of impacts on residents [19–21]. Research directly with care home managers has been small-scale and general in focus [12, 20, 22–24] and/or small numbers of care home managers have been included in wider samples [21]. With the exception of a small, mixed sample study in Scotland [21], none has looked specifically at experiences of developing and implementing visiting policies. In a report commissioned for the Scientific Group for Emergencies (SAGE), Social Care Working Group, the NIHR Older People and Frailty Policy Research Unit [17, 18] specifically identified the need for studies on visiting policies in care homes utilizing larger and more systematic samples and that take account of care homes’ wider organizational and regulatory context [17, 18].

Our study aimed to answer the question, ‘how did care home managers in England experience and respond to the national guidance on care home visiting during the Covid-19 pandemic?’ In particular, we considered the influence of the guidance itself, different care home characteristics, the role of national and local regulators, and the guidance, information, advice and support that care homes drew upon to develop and implement their visiting policies.

## Methods

We used a qualitative research design for its ability to examine the lived experiences and personal perspectives of care home managers. This involved a multi-method approach involving two inter-locking qualitative methods; [25] a qualitative survey of care home managers across England, in which respondents were asked to write short prose answers to ten questions (*n*=up to150), and follow-up, in-depth online interviews lasting 45 minutes to an hour with a purposively selected sub-sample of survey respondents (target *n*=40).

The qualitative survey was designed to generate data capturing range and diversity across a wide range of care home types and circumstances [26]. For maximum variation, survey respondents were recruited through a range of routes including Clinical Research Networks (CRNs), the Enabling Research in Care Homes (ENRICH) network, several large- and medium-sized care home providers and a range of local and regional care home networks. While participation was entirely voluntary, care homes within these different networks were pro-actively approached and invited to participate, commonly within already established and recognised structures for recruitment into research. This helped to mitigate the methodological limitations of ‘opt-in’ or self-selection processes. Care home managers were given written information about the study and what participation would involve. Those who agreed were sent a link to an online survey. The survey took approximately 20-30 minutes to complete. It could be saved and returned to later and, where the manager was not best placed to answer a question, a link enabled these questions to be forwarded to a relevant colleague. Researchers were available by email and telephone for queries. The ten survey questions are listed in Table 1. Responses were saved in a secure, online repository, where they could be accessed only by authorised researchers. The survey took place between March 2021-January 2022. Data were analysed using Framework, a theoretically and methodologically flexible tool suitable for collaboration in teams with multiple researchers [17, 27, 28], using Excel software. A Framework matrix was established for each survey question, with headings (themes) within each matrix developed inductively.

**Table 1:** Qualitative survey questions

**Question number**	**Question**
**Introductory Text**	In the survey, we invite you to write a short narrative answer for each question. This could be just one short paragraph or could be more depending on how much you have to tell us.
**1**	**What has been your experience of using the Government guidance on care home visiting (or summaries of the guidance) in developing your visiting policy?** We refer to the Government guidance originally published on 22nd July 2020 and any subsequent updates. The current guidance can be found here: https://www.gov.uk/government/publications/visiting-care-homes-during-coronavirus/update-on- policies-for-visiting-arrangements-in-care-homes#developing-the-visiting-policy-in-the-care-home
**2**	**Please tell us about any other guidance, resources, advice or support that you have used to develop your visiting policy.** These may include, for example: - guidance documents from non-Government organisations - practical tools such as flow diagrams or decision aids - professional consultancy or advice It could also include local-area protocols or frameworks, or advice from the local authority or the local Director of Public Health. In your answer, please tell us about your experiences of using these sources of support, including what you found more or less useful.
**3**	**Please tell us about any other types of support for developing your visiting policy that would have been helpful but were not available to you?** In your answer, please tell us why these would have been helpful and whether there is any support that you feel you are still lacking.
**4**	**Government guidance recommends that care home visiting policies are responsive to the needs of individual residents.** **How, if at all, is this reflected in your visiting policy? What opportunities and challenges have you experienced in developing this aspect of your policy?**
**5**	**How and to what degree have you been able to take account of the views of residents, families and staff in developing your policy?**
**6**	**What, in your view, are the key lessons to be learned from the process of developing your visiting policy?** We are interested in lessons for all or any of the following: - your care home - other care homes - Government and policy-makers - others
**7**	**How did you communicate the visiting policy to residents, families and staff? What worked well and less well?**
**8**	**How was the policy received by residents, families and staff?** a. Do you think residents found the policy easy to understand, fair and proportionate? Why/ why not? b. Do you think families found the policy easy to understand, fair and proportionate? Why/ why not? c. Do you think staff found the policy easy to understand, fair and proportionate? Why/ why not?
**9**	**What has been your experience of using the visiting policy?** What has worked well and less well? On reflection, are there aspects of your policy that you think could be improved?
**10**	**How, if at all, do you expect your visiting policy to change in future?** In your answer, please think about the short- and longer-term.

Follow-up qualitative interviews were conducted between June 2021 and February 2022 with a purposive sub-sample of survey respondents, selected for maximum variation by type of home, size of group, number of residents and geography, as well as to reflect a range of issues arising in survey responses. Only those indicating willingness to participate in a qualitative interview were contacted, initially by telephone with information and consent forms exchanged by email. If willing, an interview was arranged at a time convenient for the respondent using a video-conferencing platform of choice. A topic guide was developed based on the original research aims and insights gained through analysis of survey data. This provided prompts for exploring, in detail, managers’ experiences of developing and updating their visiting policies using the national guidance, how policies were communicated to residents, familes and staff and how policies were implemented in practice. Table 2 shows the full topic guide. This guided the interview and helped to ensure coverage but was used flexibly to allow respondents to freely discuss topics of most importance and interest to them and to enable new issues to arise. Interviews were audio-recorded with permission and professionally transcribed verbatim. Each participant received a payment of £50 for their care home’s activity fund.

**Table 2:** Topic guide for in-depth interviews

**Section**	**Discussion points**
**Preliminary instructions**	Please adapt to suit the specific care home you are planning to interview, including adding in questions arising from their survey responses. Use flexibly. It is more important to gain adequate depth on a handful of key issues than necessarily cover every possible area of inquiry.
**Introduction**	• Recap how the interview will be conducted (some topics you want to cover but conversational, how long it will last, will be recorded with permission, can stop at any time or not answer any questions they don’t want to etc.) • Recap confidentiality information from invitation letter • Ask if that is all clear and if they have questions • Ask if they are happy to proceed
**Developing and revising the visiting policy**	• Ask in detail about their process for developing their visiting policy • Did they have a written visiting policy before the pandemic? • Did they have a written visiting policy after the first lockdown but before the first Government guidance in July? How did they develop this? • Use and experience of using Government guidance produced in July 2020 and then subsequent guidance? • What other sources of information, guidance and support did they draw upon or were they required to follow? This could include guidance from other bodies, consultants, in-house specialists or, more formally, guidance from national and local Public Health England or Local Authority guidance/ advice. • How, if at all, were they restricted in developing their visiting policy? • What skills were needed? How did they access these? • If part of a group of homes, what support were they provided with centrally? • To what degree did they engage in peer learning (between care homes/ care home organisations)? • Other support they needed/ wanted or thought would have been useful? • What was important or different about their home, compared to others, that shaped how they responded in developing their visiting policy? • Capture changes over time, including the different versions of Government Guidance and regulations • What was most helpful, why? What was least helpful, why? Any gaps? • What could Government have done better in supporting them? What, if anything, did they do well? • How did they handle personalisation within their policies (i.e. adapting visiting policies to individual circumstances and need)? • What scope did they have to do so? What, if any, limitations were there? • How did they interpret the guidance on this? • Did this vary over the course of the pandemic and how? • What support, if any, did they have with developing and implementing individual risk assessments? • What was important or different about their home, compared to others, that shaped how they responded to creating policy around, and implementing, personalisation?
**Communication**	• How, if at all, did they gain input from stakeholders, including staff, residents and families and any external stakeholders. • Think about how they did this for different iterations of the policy over the time? • What made it challenging? • What opportunities were there and what worked well, and why? • What was their perception of how residents and family members viewed the trade-offs necessary between reducing the risk of transmission of the virus and reducing the risks associated with not ing visitors. • How did they communicate the policy to staff, residents and family? • Explore in detail what methods worked well and less well? • What resources were needed for this? • What skills were needed? • What were the challenges? • What support did they draw upon? • Was there any support that they did not have that would have been useful? • How did staff, residents and families respond to the guidance (discuss any variation between and within these groups and how this was handled)?
**Implementation**	• How workable was/were the resulting policy/ policies? • Any potential improvements that could have been made in retrospect? • Were there any equity considerations, in practice? • In practice, how acceptable were the policies to staff, residents and families – what trade-offs were necessary/ made? • Were there impacts of how staffing was organised or other staffing considerations?

Interview data were analysed thematically using the Framework approach with Excel software [27, 28]. Within this, our thematic analysis followed the six steps outlined by Braun and Clarke - familiarization, coding, generating themes, reviewing themes, defining and naming themes, and writing up (2006; 2022) [29, 30]. Specifically, Framework matrices and headings (codes) were developed collaboratively and iteratively, through team discussions and based on survey findings, familiarisation with transcripts and coding of a small number of initial transcripts. When all data were fully charted, central (secondary) charts were then developed. These drew data from different parts of the overall Framework to identify and develop key themes. Five researchers were involved in the analysis (JD, KLD, KHD, ES, MM) and evolving analyses were discussed at regular intervals with an advisory group involving care home managers and care home providers and an experts-by-experience group of family carers.

Reported findings draw primarily from the in-depth qualitative interview data but are augmented with selected quotes from the survey, where relevant. Findings should be understood as cutting across all or multiple versions of the guidance, except where specified or implied by context, reflecting the fact that it was generally not practical for respondents to identify and discuss specific versions of the guidance. The first mention of an organisation or term is given in full and thereafter common acronyms or abbreviations are used. A list of common acronyms and abbreviations used throughout the paper can be found at the end of the paper.

## Findings

In practice, managers from 121 care homes responded to the qualitative, online survey and 40 of these participated in a follow-up, in-depth qualitative interview. Table 3 describes the achieved survey sample and the achieved sample for the qualitative interviews, showing the diversity by type of home, size of group, number of residents and geography. The initial codes (Framework headings) for the analysis of qualititative interviews are summarised in Table 4.^7^ Central (secondary) charts, drawing from and building upon the initial charts, generated eight key themes. Findings are organised and discussed under these eight key themes, which are;

- Government guidance; issued late and not user-friendly
- Frequent, reactive and media-led updates
- Provided local flexibility vs ‘easy to interpret any way you wanted’
- Guidance and advice from local regulators and stakeholders, variable and uncoordinated
- Wider information, advice and support; valuable but duplicative, overwhelming and confusing
- Restrictive interpretations, and lack of discussion about wider risks and harms and human rights
- Insufficient recognition of dementia, end of life and other specific needs
- The importance of care home leadership and staffing

**Table 3:** Achieved samples

	**Survey**	**Interviews**
**Type of care home**		
Nursing	65	18
Residential	53	18
Both	3	4
* n*=	121	40
**Size of care home group (no. care homes in group)**		
1	53	16
2-10	26	9
11-20	19	8
21-30	3	2
31-40	4	0
41-50	0	0
51-60	0	0
61-70	0	0
71-80	2	0
81-90	1	0
91-100	0	0
101-200	5	0
201-300	1	0
301-400	7	5
* n*=	121	40
**Size of care home (no. of residents)**		
0 - 10	8	2
11 - 20	11	2
21 - 30	14	3
31 - 40	20	8
41 -50	16	5
51 - 60	17	7
61 - 70	12	3
71 - 80	8	4
81 - 90	5	0
91 - 100	1	1
100 +	9	5
* n*=	121	40
**Geographical location**		
East Midlands	6	2
East of England	22	3
London	19	8
North East	15	3
North West	13	7
South East	21	9
South West	4	3
West Midlands	15	3
Yorkshire and Humber	6	1
Multiple areas	0	1
* n*=	121	40

**Table 4:** Framework matrices and headings (codes) for initial charting and analysis of qualitative interviews

	**Pre-Covid**
1.1	Pre-pandemic perspectives/ ethos on visiting
1.2	Pre-pandemic visiting policies/ arrangements
	**Views on Govt guidance**
2.1	Too vague, left too much to discretion vs. too directive
2.2	Easy or difficult to read, clarity
2.3	Timing, when guidance was issued and changes over time
2.4	Specific content
2.5	Copy and pasting sections of guidance, using guidance as template etc
2.6	Whether accommodates care home variability
2.7	Other
	**Other guidance, information and support to develop policy**
3.1	Internal resources (within the company)
3.2	Commissioned (bought in) services and support
3.3	Care homes learning from each other (forums/ WhatsApp etc)
3.4	Local Authority
3.5	CQC
3.6	Public Health England (PHE) Health Protection Teams (HPTs)
3.7	Other agencies/ bodies
3.8	How advice and support all fitted together
3.9	Other
	**Other issues influencing development of visiting policies**
4.1	Visiting policies perceived as more or less restrictive than Government guidance
4.2	Human rights aspects
4.3	Insurance and legal aspects
4.4	Care home ownership
4.5	Testing
4.6	Vaccination
4.7	Role of media/ media announcements
4.8	Care home/ sector consulted/ not consulted by Government and others
4.9	Resource implications
4.10	Other
	**Staffing**
5.1	Consultation with staff
5.2	Communicating policy to staff
5.3	Impacts on/ responses of managers and staff
5.3	Other
	**Personalised visiting**
6.1	How to assess risk for individuals (general)
6.2	Visits for immobile/ bed-bound residents
6.3	Visits for people with dementia
6.4	Visits for people at end of life
6.5	Essential caregivers
6.6	Other
	**The future**
7.1	Returning back to the 'old' normal
7.2	Finding a 'new' normal
7.3	Vaccination and other future developments that may influence ability to visit safely
7.4	Flexibility, uncertainty
7.5	Other
8.1	**Analytical comments**

### Government guidance; issued late and not user-friendly

Managers in smaller groups or stand-alone homes were generally familiar with, and able to discuss, the national care home visiting guidance, although they sometimes found it difficult to clearly distinguish between different documents they had used when developing their policies. In larger groups, where visiting policies were centrally developed, managers were less likely to have read the national guidance although most had read at least a summary.

Most managers were aware that the initial guidance was not issued until Summer 2020. Some thought this understandable given that the pandemic was *‘an unknown situation’* and said, *‘the Government has moved quickly in a number of areas and should be applauded for that.’* More commonly, however, managers described it as *‘slow, incredibly slow,’* sometimes reflecting that Government was *‘not really there for us.’* This initial lack of guidance was commonly experienced as *‘very stressful.’* Managers described having to *‘read everything in sight and use instinct.’* By the time the guidance was issued, some also saw it as *‘lagging behind’* and not telling them anything they didn’t already know.

Care homes were ahead of Government guidance, so when it came out it was teaching them to ‘suck eggs’ (Interview, South East).

Some thought the guidance was the best that could be expected in difficult circumstances.

They were trying to give us the best guidance at the time from their experience. I do feel that, you know. (Interview, North East)

Others described the guidance as ‘*straight-forward’* and ‘*clear what the care home is expected to do to.’* However, more commonly, managers thought it was *‘lengthy,’ ‘cumbersome*’ and *‘unclear to follow.’* It was also described as having multiple *‘gaps,’* with one manager commenting that *‘it feels that part of the information is missing.’* Many noted that reading and interpreting national guidance was ‘*not normal’* for them (although there were exceptions, e.g., one manager had experience of NHS emergency planning), and, for those from smaller groups or stand-alone homes especially, it was a *‘huge administrative burden.’*

Managers made multiple suggestions for making the guidance more user-friendly, including simplifying it and focusing on practical actions.

Actually, what I would have liked is a very simplified ‘at this moment this is what we allow, this is what we…’ (Interview, London)

Specific suggestions included ‘key points summaries’ and an ‘easy to follow flow chart,’ as well as materials to share with residents and families such as ‘an A4 page to put up in [the] care home’ and ‘easy to understand information for people living with dementia.’ Others suggested ‘basic paper-works’ such as sample documents or standard templates for policies, risk assessments and letters; ‘you can use these or not, but at least it is there and it fits with what’s being asked’.

### Frequent, reactive and media-led updates

Managers commonly learnt about frequent updates to the guidance through televised Government announcements, often on Friday with written guidance not available until days later. This put managers *‘on the back foot’* and made it difficult to implement changes in a timely way. Measures such as risk assessments, visiting screens or pods and testing areas were specifically mentioned as difficult to implement quickly. Managers also lacked time to train and prepare staff, which could result in confusion for staff and families and raise concerns about safe practice. Regulators were sometimes equally surprised by Government announcements and took time to issue their own local advice and guidance.

I rang Public Health England and said, ‘well, what’s happening?’ They said, ‘we don’t know, we only heard it on the news last night.’ (Interview, South East)

Having changes announced in the media could also lead to families having ‘unrealistic expectations.’ For example, one manager described families who had found out about ‘essential caregivers’ from a televised Government announcement, not understanding that the role carried obligations and requirements. This could cause anxiety and ‘more work’ for managers. Managers called for ‘more consistency from central Government to ensure that interviews and press releases reflected what was actually going to be possible’ and some emphasised the onus it put on them to communicate clearly with families.

I think you’ve got to be strong enough to say, ‘no, actually, we haven’t had any formal guidance, we’re waiting for guidance from the government, proper guidance, from our local authority and as a group of people we will discuss how we go about this as a group.’ (Interview, West Midlands)

Managers also commonly found it *‘challenging keeping up with the volume of information released’* and were not always clear on how to find changes in the guidance, although some managers reported clearer bulleting of key changes over time.

They start off by mentioning the amendments that they’ve made now, so you know exactly where to go to (Interview, South West)

Exceptionally, managers welcomed frequent updates so visiting policies could be adjusted to reflect new information and circumstances. Some also said that they had become *‘used to’* or *‘better at’* keeping up with the legislation and guidance. One said, *‘I used to print it out, now not, just dash through it.’* Others said that they didn’t wait for the guidance at all, since it was time-consuming and sometimes unclear, but relied solely on televised announcements.

TV was useful as it was more succinct than the written guidance which was very wordy and lengthy (Survey response, East of England)

### Provided local flexibility vs ‘easy to interpret any way you wanted’

The Government guidance, and subsequent updates, were widely considered *‘open to interpretation.’* Some thought this was so that visiting policies could be adapted to local needs and circumstances. However, they sometimes conceded that this intention *‘was hidden so much in the policies from Government, that some people didn’t read it.’* These managers viewed the guidance as *‘a good starting point’* or *‘useful foundation’* and thought managers should be more confident in developing visiting policies suited to their local circumstances, commenting, *‘at the end of the day, they are only guidelines.’*

If there are parts that we either cannot follow, or we feel are inappropriate for our circumstances, we make the decisions on what we can do/will do (Survey response, North West)

More commonly, however, managers viewed the guidance as *‘vague’* or *‘woolly,’* saying there was *‘too much for care home managers to decipher,’* and that it was *‘ambiguous’* and *‘easy to interpret it however you wanted.’* Some feared that this could encourage *‘a postcode lottery’* of different visiting rules and restrictions. Managers also found it difficult to generate practical actions using the guidance, stating that it did not *‘offer any solutions for providers.’*

There are gaps, things that are not covered in the guidance, and we’ve struggled to develop workable practical policy from the guidance (Survey response, South East)

Managers also noted a lack of discussion in the guidance about different care home contexts, stating that *‘the Government were treating all care homes the same when they should be treated differently.’* This was often thought to reflect a lack of practical understanding of the sector, with managers commenting that you *‘could tell it was written by people who think they might know how we work’* but who *‘don’t really know care homes.’* While the guidance required managers to develop risk-based visiting policies, it also offered little advice about how to assess the multiple risks involved.

You create this risk assessment based on a virus that we, as the Government, don’t know enough about yet. We don’t know the impact it’s going to make, but you create it, and create it without a policy or guidance. (Interview, South East)

These various gaps led some to feel that responsibility for policy on care home visiting had been unfairly shifted on to care home managers and groups.

### Guidance and advice from local regulators and stakeholders, variable and uncoordinated

Managers commonly found it difficult to obtain a clear or unified view of local regulators’ requirements and expectations with regard to visiting policies. They described a lack of coordination and of having to negotiate *‘a series of local guidances’* that were sometimes unaligned, conflicting or appeared inconsistent with Government guidance. Managers could find this overwhelming and confusing.

CQC (Care Quality Commission), Skills for Care, Public Health England, your local resilience team, your clinical lead, National Care Forum, and in the end, you were going, ‘well, actually which guidance are they all referring to. (Interview, South East)

Consequently, managers found that they had *‘to do research about local policy’* and liaise with regulators to resolve uncertainties and differences of interpretation with each new change in the national guidance. A suggestion was made that the national guidance and subsequent updates should have been agreed with regulators nationally to help ensure consistent local interpretations.

Support from local regulators varied considerably. Local authorities had wide-ranging responsibilities for care homes as part of the national Covid-19 emergency response; as commissioners, because of statutory responsibilities for social care and other services, due to their responsibilities under the Coronavirus Act 2020 and as a result of their role on local resilience forums. Consequently, they offered some of the most comprehensive and responsive support, particularly during the initial stages of the pandemic when visiting was highly restricted. This included daily check-in calls, telephone advice lines, facilitated managers’ groups, feedback on risk assessments and provision of equipment such as tablets. Managers often described this support as invaluable and ‘*a light in the dark.’* However, the quality and reach of support varied by area and type of home, with managers of homes without local authority-funded clients sometimes reporting not being contacted by the local authority or of *‘being at the bottom of the pile’* for receiving support. Occasionally, managers described the level of contact with the local authority as excessive, especially later on in the pandemic.

They rang us every single day … It was very positive, very supportive. Latterly, as we progressed through, it became a bit of a pain to be honest because there was nothing else I had to tell them. So, sometimes it was just, you know, they’d be telling us generally about them, and I’m like, ‘I haven’t really got the time’. (Interview, North East).

The Care Quality Commission (CQC), in contrast, were widely characterised as *‘absent’*. One manager drew a comparison with the comparable inspectorate in Scotland (Care Inspectorate), saying that it had established a 24-hour helpline and redeployed inspection teams to help in care homes whereas, *‘ours sat and were mute for months.’* Many managers consequently saw the CQC as a potential threat, more likely to criticise their visiting policy than support them to develop it. Some also saw CQC as inconsistent and reactive, initially giving the clear message that care homes should not allow visitors and then, later, blaming them for implementing ‘blanket bans.’

CQC withdrew from the homes. The message was homes are not open. And they changed their tone across the course of the pandemic, their positioning changed, not steady and working alongside the sector. (Interview, South East)

However, some reported supportive relationships with their inspectors who remained on hand to offer advice. One, for example, described an inspector spending an hour on the phone with her *‘because [she] had some Covid deaths, and was getting worried.’* Some were grateful that CQC paused inspections; *‘they sort of said, ‘right, well, we’re not visiting, we’ll keep out of your way, let you get on with it,’* however, managers also described feeling relieved once CQC began inspections again (April 2021 onwards) and they had received a positive inspection outcome.

Contact with local Health Protection Teams (HPTs) was relatively uncommon outside of a Covid-19 outbreak. However, sometimes, managers contacted their local HPT for advice about visiting arrangements. A number described only being able to reach a *‘call centre’* with unqualified staff, while others said that their HPT was supportive, *‘depending upon who picked up their phone’*. Where there were outbreaks, some HPTs were experienced as ‘*understanding and helpful’* and *‘fantastic during the worst time.’* However, some described them as *‘project managers’* and *‘not specialists,’* as lacking practical experience of care homes, giving impractical advice and being *‘uncompromising’* and *‘inflexible.’*

Managers also said that health professionals withdrew physically from care homes. However, there were examples of ongoing close support from Clinical Commissioning Groups (CCGs), particularly where there were existing strong individual relationships. Infection control training provided through the NHS was widely considered unnecessary, patronising and as *‘teaching [them] things they already knew.’*

### Wider information, advice and support; valuable but duplicative, overwhelming and confusing

Wider sources of information, advice and support included that provided by care home groups, external organisations and various forms of peer support. Typically, larger care home groups had dedicated central, and sometimes regional, staff to support managers by developing visiting policies centrally, facilitating managers’ meetings and assisting with implementation, legal and regulatory issues. Managers of stand-alone homes or homes in smaller groups were more reliant on external support. However, they sometimes saw not having a visiting policy *‘imposed by a bigger organisation’* as a benefit and felt more able to generate *‘bespoke solutions’* and *‘react quickly to changes.’* Occasionally, managers purchased standard policies from commercial suppliers. These were valued for their convenience and regular updating, but some thought they needed a lot of editing, restricted flexibility and that it was inappropriate in a pandemic to be ‘*waiting for a company to tell you what to do.’*

Many external sources of information, advice and support were found valuable. For example, respondents mentioned Department of Health and Social Care (DHSC) webinars and support from Skills for Care, the Social Care Institute for Excellence, Care England, the Care Provider Alliance, National Care Forum, Healthwatch, Dementia UK and the Alzheimer’s Society, as well as various residents and families’ lobby and campaign groups and commercial support including independent consultants and resources such as a software company that had *‘put the hefty guidance into simple easy to follow guides.’* However, managers said that, while such support was initially lacking, later, they were *‘deluged with information’* with ‘*too many people doing the same job.’* Support was also often experienced as uncoordinated, time consuming to engage with, duplicative, confusing and contradictory.

Sometimes it just doesn’t match up. I just think everybody’s jumped on the bandwagon. I know they’re trying to help, but I think it’s too much. (Interview, West Midlands)

Managers also engaged in peer-based support groups, using video-conferencing software (e.g. Zoom or Teams) or asynchronous digital platforms (e.g. Facebook or WhatsApp). These were sometimes instigated and facilitated by care home groups, local authorities or national membership organisations. In other cases, they developed from existing managers’ networks. As well as sharing information and talking through problems, members of such groups sometimes shared documents and policies as well as practical tasks, such as checking guidance or watching and reporting back on the televised Government announcements. Managers in large care home groups appreciated gaining a *‘wider view’* by being involved in area- or sector-based groups. The groups also provided managers with a sense of camaraderie and emotional support.

I couldn’t have done it all by myself, so by having that group there for support, and being able to take other people’s ideas and suggestions, and make it work for us. Without that I would have felt very much on my own. (Interview, North West).

However, some experienced peer groups as *‘not constructive’* or described meetings in which managers *‘were struggling,’ ‘just moaned,’* shared bad practice or had disagreements. One manager, for example, described others in a peer-based group as *‘a bunch of idiots’* because she did not feel they were taking infection control seriously. Well-facilitated groups were especially valued, however, as well as groups where senior staff or experts were available to address questions such as at some local government-run fora and a large WhatsApp group in which authoritative and *‘up-to-date information’* was provided by leading national academic clinicians.

### Restrictive interpretations, and lack of discussion about wider risks and harms and human rights

Government guidance on visiting was commonly seen as prioritising infection control, with significantly less discussion about other risks and harms to residents’ physical, mental and emotional well-being or human rights. This focus was sometimes welcome, particularly earlier in the pandemic, with some saying that they *‘made no apologies’* for keeping their ‘*home and staff safe.*’ However, as the pandemic progressed, others thought that Government was *‘privileging physical health and forgetting the emotional, social and spiritual needs of residents’* and ‘*the effect on people as human beings.’* These managers saw themselves as expected to implement restrictive, even inhumane, measures without the support of a clear framework for considering wider risks and harms, and human rights.

This was compounded by restrictive interpretations of the guidance by insurers and local regulators. Some managers seeking to implement a risk-balanced approach described themselves as ‘*over-ruled’* by regulators, who *‘have their own interpretation’* and who *‘appeared to be taking the guidance as statutory, which is confusing and leaves responsible people unclear how to use the guidance.’*

You’d go on the Government website, and it would say, ‘but it is at the home’s discretion’ and you think well, ‘you’ve [Health Protection Team] just told us it’s a legal requirement. So, is it, or isn’t it?’ (Interview, South West)

Other managers described being made to feel ‘*reckless’* or *‘maverick’* if they adopted less restrictive visiting policies and felt under pressure to follow regulators’ ‘advice.’

We have been in a cold, hard place with the visiting. I think Government have been very diplomatic putting a lot of stress on the managers, but [the Health Protection Team] say they advised you not to do that and, at the same time, you’re governed by [Care Quality Commission]. So, if we do not follow the advice, then we are breaching the regulations. (Interview, South East).

These managers thought they would be blamed for any outbreak; a standard that seemed unreasonable. Some described it as ‘*very much a blame game,’* saying *‘it felt like, ‘ideally do this, but if you’don't do this then we will blame you.’* This perception persisted despite changes in some bodies’ public positioning regarding ‘blanket bans.’

[Care Quality Commission] made a statement outlawing blanket bans. Well, we all agree with that, until it goes wrong, then we feel like, ‘oh shit’ we're going to get blamed.’ (Interview, South West)

Specifically, managers were concerned about potential legal and financial implications, reputational damage and negative effects on their *‘long term position’* and relationships with regulators. There was a widespread perception that similar pressures were felt by larger care home groups.

So, ’that's why a lot of care providers went, ‘okay, regardless of what the policies say for visiting, shut down completely’, and they were very reluctant to open, but it was fear. (Interview, South East)

Managers sometimes regretted succumbing to these perceived pressures and *‘in hindsight’* wished *‘they had allowed more’* visiting. Commonly they thought there should have been greater ‘*clarity on some core mandatory requirements of the visiting guidance and areas where [they] could have flexibility without fear of reprimand,’* as well as ‘greater emphasis’ on, and support for, *‘human rights,’ ‘risk assessment and person-centredness.*’

### Insufficient recognition of dementia, end of life and other specific needs

Managers felt that the specific needs of sizeable numbers of residents were insufficiently addressed in the guidance. This included people with dementia, with many noting that *‘dementia appears to have been ignored.’* Although, initially, some residents with dementia responded positively to the quieter environment of a home without visitors, overall, residents with dementia were thought particularly vulnerable to the loss of family visits and usual routines, with impacts including loss of appetite, apathy, distress, confusion and depression, and increased risk of falls. Despite this greater vulnerability, managers found many aspects of the guidance to be impractical or unethical for people with dementia. They noted that *‘people with dementia’don't understand that there is a pandemic’* and cannot voluntarily observe restrictions. Residents with dementia also often struggled with digital communications, even with support, while window and screen visits frequently caused confusion and distress, and, for in-person visits, managers said that a *‘lack of hugging or cuddling is not just inhumane but practically unmanageable.*’ Managers were required to enforce self-isolation during outbreaks or following hospital visits or advised to move or cohort residents with dementia. These measures were commonly seen as impractical for people with dementia, resulting in distress, depression and risk of falls. One manager said, ‘*Government ministers should not issue blanket initiatives which may not be feasible.’*

You’can't isolate people with dementia. It’s unethical and it’s morally and everything wrong, so you can’t do it. (Interview, South East)

In response to these challenges, where staffing allowed, residents, particularly with dementia, were kept company by staff and engaged in activities. Some even reflected that *‘unlike a lot of the elderly community, they were never isolated, they always had company here even if it wasn’t their family and friends, there was always someone to talk to. They had each other. They had us.’* Facebook pages were sometimes used to share photographs of these activities with families.

I’d put photos on there of activities and how life is very much continuing, and you know people are still okay and they’re still happy and they’re still doing quizzes and singing and all the things you would expect them to do. (Interview, South East)

Where residents with dementia experienced physical or mental deterioration, managers facilitated compassionate visits or ‘*turned a blind eye’* to physical contact during in-person visits. Managers also sometimes adopted less restrictive visiting policies; while open to challenge by regulators, this became more possible following the introduction of testing, vaccination and essential caregiving. Managers also directly challenged regulators when asked to implement measures that, in their view, were impractical or put residents at risk.

She said, ‘well this is what you’ve got to do,’ and I was kind of disagreeing with her and saying, “I understand that legally this is the only guidance they’ve put out, and I know that you’re relaying that guidance to me, what I’m saying to you is that’s not going to work.’ (Interview, North West)

End-of-life visiting was also thought insufficiently discussed in the guidance. Managers sometimes said that the definition of end of life and what was allowed during an end-of-life visit were unclear. This could lead to varied practices. During lockdown, some homes did not allow end-of-life visits at all or only in the last days or hours of life, with varied approaches to wearing PPE and touching, while others permitted visits in the last weeks of life. However, others adopted intentionally wide definitions to facilitate visits. For example, one manager commented, *‘If they are bed bound and can’t get out for a garden visit, then they obviously are not well.’* Regulators, however, sometimes adopted even more restrictive interpretations than managers.

In [the inspector’s] view it said, ‘end of life’, so he was reading the guidance black and white, so ‘end of life,’ and these people obviously hadn't died. (Interview, multiple areas)

Restrictive definitions were considered problematic given challenges in identifying end of life. Consequently, in practice, we found that managers sometimes called families in for compassionate visits with the resident later recovering or, conversely, failed to recognise that residents were at end of life until too late.

We had to just assess when we thought and there was a few that we missed that opportunity which was quite sad. (Interview, East Midlands)

Ethnic and religious diversity were also little discussed in the guidance although were sometimes relevant in visiting decisions. For example, in one home an Asian resident was permitted food brought in by her son and one manager discussed the specific challenges in implementing isolation policies for residents in a Jewish care home, many of whom were survivors of Nazi concentration camps. Also, while managers said that many families found identifying a single or limited number of constant visitors challenging, this was even more difficult in larger families, including amongst certain ethnic or religious populations.

### The importance of care home leadership and staffing

The circumstances of the pandemic placed unprecedented demands on managers and staff. Managers described working extremely long hours, not seeing their own families, losing sleep, and experiencing moral distress, anxiety, depression, exhaustion and burn-out.

In all this visiting, it’s up to me to play God. That’s how it feels. It’s all up to the manager to make those final decisions. (Interview, London)

Managers also varied in their leadership skills and experience and those with previous clinical experience (for example, working as nurses with experience of managing Norovirus or MRSA, or, in one case, working in NHS emergency planning for SARS) may have fared better. Staff were similarly described as often working long and flexible hours. and going to lengths to limit their own exposure to Covid-19 outside of the care home. They were also expected to rapidly implement new visiting systems and policies with limited training or preparation, and managers described staff fearfulness, moral distress, burn-out and exhaustion.

I think that’s why a lot of staff have suffered burn-out; lots of staff have left the sector because there’s things we’ve had to do that just don’t fit into either our ethos or what our job role is. (Interview, East Midlands)

Some, but not all, managers had been able to offer staff, especially those that had experienced significant resident deaths, counselling and well-being support. Significant under-staffing was also common, increasing stress for managers and remaining staff.

I understand why [manager] actually left. I think it was all stress related, because some days she didn’t even know if she’d have one staff member turn up. (Interview, South East)

Managers sometimes drew on bank or agency staff but thought them more likely to make mistakes. In some cases, volunteers helped with, for example, visitor testing and escorting. Understanding of the essential caregiver role by managers, insurers and regulators varied, as did family carers’ enthusiasm for adopting the role. However, some managers designated as many family carers essential care givers as possible to both facilitate visiting and ease pressures on staff. While testing and vaccination supported these developments, sometimes managers *‘wish[ed they] had had volunteers and essential care-givers throughout.’*

## Discussion

We aimed to identify how care home managers in England experienced and responded to national guidance on care home visiting during the Covid-19 pandemic. The picture we found was complex. The guidance, eagerly awaited by care home managers and the sector as a whole, was published in July 2020, approximately three months after the initial national lockdown. Some were positive about the guidance. They either viewed it as supporting the restrictive measures they felt necessary to protect residents and staff from infection or saw it as setting a broad policy framework while allowing for local discretion. However, more commonly, managers identified multiple challenges.

While larger care home groups had policy staff, many users of the guidance were operational managers. Shorter, more practically-orientated guidance, potentially with locally-adaptable elements such as templates, flow diagrams, brief assessment tools and information to share with families, could have helped to reduce the burden on managers, particularly in smaller care home groups and stand-alone homes, limited ‘reinvention of the wheel,’ and supported more timely and effective implementation. Updates could also have been more helpfully communicated. These were usually announced in the media, often on Friday, with written guidance provided sometimes days later. This generated confusion and anxiety for staff and families, created considerable work for managers and made implementing changes within expected timeframes difficult. Positively, however, managers thought that, over time, signposting of key changes improved. Managers also became better at navigating the guidance, although some stopped reading it altogether, relying instead solely on media announcements.

The content of the guidance was widely described as *‘open to interpretation’.* Exceptionally, this was thought intentional, to allow for local discretion. However, others pointed to important gaps, notably concerning the ethical acceptability and feasibility of social distancing, isolation and visiting restrictions for residents with dementia and the challenges of defining and identifying end of life and managing end-of-life visits. These gaps were difficult to understand given that most care home residents have dementia and limited life expectancy [31, 32]. Managers sometimes understood this as evidence of Government’s poor understanding of the sector, which has been frequently noted by commentators [10, 33]. These gaps left managers not knowing how to apply the guidance in practice. This echoes similar commentary concerning discharge of NHS patients into care homes at the beginning of the pandemic, with Devi et al. noting *‘a paucity of useful guidance,’* which was *‘silent’* or *‘ambiguous’* on important operational issues [34], and Martin Green (Care England), in evidence to a parliamentary committee, describing the guidance as *‘not really connected to the reality of lots of care homes’ *[2].

More generally, despite requiring a *‘dynamic risk assessment,’* the guidance was seen as having little to say about how the risks and harms of visiting restrictions should be assessed or mitigated. The guidance was also seen to lack clarity about how fundamental human rights protections, including the rights to life (Article 2, ECHR),^8^ liberty (Article 5 ECHR) and a family life (Article 8 ECHR), should be upheld and balanced. These gaps were of immediate practical concern to managers, with many left feeling exposed, conflicted and vulnerable to moral distress. There has since been widespread discussion of these issues, e.g. in expert commentaries [12, 13, 16], in a report from the Parliamentary Joint Committee on Human Rights [10] and in the context of a prospective legal challenge from the UK charity, John’s Campaign [35].^9^ A widely noted lack of pandemic preparedness, particularly in social care settings, may have contributed to the lack of a more developed framework for considering these issues [36]. In terms of implications for research, drawing lessons from care home managers’ and staff’s practical experiences will be important for helping to build an evidence base concerning how the harms and risks associated with visiting restrictions can best be assessed and mitigated, and various rights balanced [5, 37, 38].

Concerns about risks and harms were compounded by the tendency of regulators to adopt restrictive interpretations of the guidance, sometimes even in the face of immediate and serious concerns for residents’ broader physical and mental well-being. Managers were often unclear about accountability in these circumstances and feared being blamed [10]. Our findings reflect those of Mitchell et al [6]. who, in an early study of care home resilience during the Covid-19 pandemic involving interviews with 10 care home managers in the East Midlands, found that *‘care home managers felt that they were held accountable by regulatory agencies for the safety of their residents, while also being expected to follow general guidelines inappropriate for their settings.’* Adding to this complexity, managers could also struggle to gain a clear view of local requirements, with multiple and sometimes apparently conflicting local sets of guidance. National and local requirements also appeared poorly coordinated, with local guidance not always clearly aligned with national guidance, and local regulators sometimes appearing to be as ‘wrong-footed’ by national policy developments as care home managers. This lack of coordinated response sometimes appeared to leave regulators lacking necessary flexibility or an adequate framework to respond effectively to the often more complex and varied circumstances they found on the ground. These findings underscore the urgent need to address long-standing concerns about fragmented systems of governance and poor central-local coordination in adult social care [33].

The use of wider sources of information, advice and support varied considerably. Managers from care homes in larger groups received support from national and regional offices, although could feel more constrained. Local authorities offered some of the most comprehensive and valued initial support. However, quality and reach varied and smaller homes and those without local authority clients reported receiving less support. Advice from other local regulators was commonly difficult to access and sometimes perceived as unhelpful, with effective support often dependent on pre-existing relationships or on *‘who picked up the phone.’* Many independent and sector-specific organisations offered highly valued support. Overall, however, there was also poor coordination, duplication and sometimes conflicting or confusing advice. These findings highlight long-standing concerns about the lack of established communication channels with reach across the sector [39]. Managers especially valued well-facilitated peer exchange and being able to interact with, and ask questions of, authoritative voices with first-hand knowledge of the sector. Strategic engagement and support from Government to help establish and promote these types of networks is likely to be helpful. There is also a question as to whether, in the circumstances, larger care home groups could have been supported to offer assistance to smaller groups and stand-alone homes.

Aside from information and advice, however, managers were often most in need of hands-on support. They commonly worked long hours, with their care homes sometimes chronically under-staffed because of existing workforce pressures, Covid-related absences and, sometimes, staff leaving because of moral distress [6, 40–44]. While staff cannot replace families [45], adequate staffing levels appeared to go a long way to averting some of the most negative outcomes for residents. For some managers, volunteers (e.g., to escort visitors and help with testing) and essential care givers proved invaluable. There was a view that volunteers could have been used more widely and the essential caregiver role established earlier to help mitigate staffing pressures [6, 10]. Research should be undertaken with managers who involved volunteers and essential caregivers, particularly earlier on. This would help to clarify the potential benefits as well as point to possible challenges and how these might be best managed. Managers with strong leadership skills, stable teams and previous clinical experience also appeared to fare better. Findings emphasise the importance of addressing on-going workforce challenges in sector, particularly through establishing parity of esteem with the NHS and a clear career structure [43, 44].

### Strengths and weaknesses

The study benefits from drawing upon a broad-based and large sample of care homes, pro-actively recruited through multiple sources across England. Trustworthiness and reflexivity were enhanced through method triangulation, [46] with qualitative methods selected for both breadth (what Braun et al., [26] refer to as a ‘wide-angled lens’) and depth of analysis. It was also enhanced through the use of multiple researchers, the use of transparent and readily shared analytic tools to support team working and the active involvement of advisors and experts-by-experience. This helped to ensure that assumptions were readily challenged and that alternative interpretations were considered [29]. Although, we recruited widely, care homes that were not part of targetted networks or felt less able to participate may, however, have had different experiences. While our findings address managers’ perceptions of other organisations and professionals, it was outside the study’s scope to speak with these directly and so their, potentially different, perspectives are not reflected in our findings.

## Conclusion

Developing policy and guidance for care home visiting, beyond relatively short periods of national lockdown, was undoubtedly extremely difficult. Care home residents are more vulnerable to severe outcomes from Covid-19 infection but also live in congregate settings, where transmission is harder to manage, so additional protective measures are merited. However, while external family visitors may increase the risk of transmission, there are potentially serious competing risks and human rights limitations associated with prolonged visiting restrictions; and a relevant evidence base to guide policy-makers was almost completely lacking. This evidence base remains sparse and is still largely comprised of rapidly conducted research using convenience samples. To ensure future preparedness, it is vital that the opportunity of the Covid-19 pandemic is taken full advantage of and that this evidence base is developed by drawing upon the practical learning of care home managers and staff. Research should adopt a rights-based approach to help identify, not just challenges and failures, but, critically, what, if anything, worked best to help maintain the human rights and well-being of residents, families and staff, in the context of such challenging circumstances.

The care home sector is complex, diverse and fragmented, and can be difficult for policymakers to engage with. However, managers commonly reported multiple and serious challenges in using the Government’s visiting guidance to develop their policies. Underlying many of these challenges were existing structural issues for which there have been longstanding calls for investment and strategic reform, calls that have remained largely unaddressed by consecutive Governments [29]. These include a need for communication channels with effective reach across the sector, less fragmented systems of governance and improved central-local coordination, solutions to ongoing workforce challenges, engaging the care home sector more fully and dynamically in the development of policy and guidance [2, 6] and establishing a comprehensive minimum national dataset [47]. These inherited challenges had negative implications for sector resilience during the pandemic. This is reflected in the findings from our study and echoed in wider discussions about the challenges facing care homes, and adult social care more generally, in responding to the Covid-19 pandemic [2, 6, 33, 36]. There were, nonetheless, also opportunities, arguably missed, to use the publication of national visiting guidance to increase clarity and coordination, reduce the practical and emotional burden on care home managers and staff, and support managers, in partnership with regulators, to evolve and implement solutions on the ground.

## Data Availability

The datasets generated and/or analysed during the current study are not publicly available, due to them containing information that could compromise the research participants’ terms of privacy and consent but are available from the corresponding author on reasonable request.

## Abbreviations

UK: United Kingdom
DPH: Local Director of Public Health
CCG: Clinical Commissioning Group
HPT: Health Protection Team
PPE: Personal Protective Equipment
SAGE: Scientific Advisory Group for Emergencies
NIHR: National Institute for Health Research
CRNs: Clinical Research Networks
ENRICH: Enabling Research in Care Homes Network
CQC: Care Quality Commission
NHS: National Health Service
DHSC: Department of Health and Social Care
MRSA: Methicillin-Resistant Staphylococcus Aureus
SARS: Severe Acute Respiratory Syndrome
ECHR: European Court of Human Rights

## Footnotes

1 See https://web.archive.org/web/20200316125115/https:/www.gov.uk/government/publications/covid-19-residential-care-supported-living-and-home-care-guidance/covid-19-guidance-on-residential-care-provision

2 See https://careprovideralliance.org.uk/coronavirus-visitors-protocol

3 See https://www.bgs.org.uk/resources/covid-19-managing-the-covid-19-pandemic-in-care-homes

4 Replaced by integrated care systems (ICS), consisting of integrated care boards (ICB) and integrated care partnerships (ICP) https://www.england.nhs.uk/integratedcare/resources/key-documents/

5 Replaced by UK Health Security Agency

6 COVID-19 supplement to the infection prevention and control resource for adult social care https://www.gov.uk/government/publications/infection-prevention-and-control-in-adult-social-care-covid-19-supplement/covid-19-supplement-to-the-infection-prevention-and-control-resource-for-adult-social-care. Last updated August 2022.

7 Two further matrices were used to compile data about communications with family carers and residents, which are not reported here

8 European Convention on Human Rights

9 Later withdrawn in light of updates to the Government guidance
